# Research productivity of radiation therapy physics faculty in the United States

**DOI:** 10.1002/acm2.13456

**Published:** 2021-10-25

**Authors:** Sanford L. Meeks, Michael H. Shang, Twyla R. Willoughby, Patrick Kelly, Amish P. Shah

**Affiliations:** ^1^ Department of Radiation Oncology Orlando Health Cancer Institute Orlando Florida USA

**Keywords:** academic productivity, bibliometrics, h‐index

## Abstract

**Purpose:**

Research productivity metrics are important for decisions regarding hiring, retention, and promotion in academic medicine, and these metrics can vary widely among different disciplines. This article examines productivity metrics for radiation therapy physicists (RTP) in the United States.

**Methods and materials:**

Database searches were performed for RTP faculty at US institutions that have RTP residencies accredited by the Commission on Accreditation of Medical Physics Education Programs (CAMPEP). Demographics, academic rank, number of publications, academic career length, Hirsch index (h‐index), m‐quotient, and history of National Institutes of Health (NIH) funding as a principal investigator (PI) were collected for each RTP. Logistic regression was performed to determine the probability of academic rank as a function of h‐index and m‐quotient. Statistical tests used included the Wilcoxon ranked sum test and the Pearson *χ* ^2^ test.

**Results:**

A total of 1038 faculty and staff were identified at 78 institutions with CAMPEP‐accredited residencies. The average RTP academic career duration is 13.5 years, with 46.7 total publications, h‐index of 10.7, and m‐quotient of 0.66. Additionally, 10.5% of RTP have a history of NIH funding as a PI. Large disparities were found in academic productivity of doctoral‐prepared physicists compared to those with a terminal master's degree. For differences in junior and senior faculty, statistical tests yielded significance in career duration, number of publications, h‐index, and m‐quotient. Gender disparities were identified in the overall distribution of RTP consistent with the membership of the American Association of Physicists in Medicine. Further gender disparities were found in the number of doctoral‐prepared RTP and physicists in senior faculty roles.

**Conclusions:**

This manuscript provides objective benchmark data regarding research productivity of academic RTP. These data may be of interest to faculty preparing for promotion, and also to institutional leadership.

## 1 INTRODUCTION

Objective metrics for evaluating research output are desirable for the assessment of faculty members.^1^, ^2^, ^3^, ^4^ Historically, research productivity has been assessed using metrics such as total number of peer‐reviewed publications or total number of citations. While these quantify research productivity, the absolute number of publications tells little regarding the quality of the publication record, and the total number of citations can be skewed by a single major publication. Alternatively, the Hirsch index (h‐index) quantifies both the productivity and citation impact of a scientist's publications.^5^ Simply put, an author's h‐index is defined as the author having published h papers that have each been cited at least h times.^5^, ^6^

The h‐index can vary widely across academic disciplines; smaller scientific disciplines have fewer scientists, fewer publications, fewer citations, and correspondingly, lower h‐indices than larger disciplines. Furthermore, clinical scientists often have clinical duties in addition to fulfillment of their academic mission, and these clinical scientists typically have lower h‐indices than those in pure science who may have primarily research and teaching responsibilities. It could be inequitable to compare academic output for faculty in clinical science to metrics for faculty in pure science, and therefore, it is important to understand benchmarks for specific subfields before using these metrics to quantify an individual's academic productivity. Hirsch noted that for researchers in traditional subfields of physics a value for h of 12 is typical for promotion to associate professor at major US research universities, and a value of 18 is typical for promotion to full professor.^5^ Multiple studies have been performed examining bibliometric benchmarks for various medical specialties.^4^, ^7^, ^8^, ^9^ A meta‐analysis was performed in 2020 that included data on 14 567 academic physicians.^10^ In this study, it was shown that the h‐index increases with academic rank with a mean h‐index of 5.2 for assistant professors, 11.2 for associate professors, and 20.8 for full professors. Of particular interest, several studies have examined academic productivity benchmarks for academic radiation oncology departments.^1^, ^2^, ^11^ The most recent study found that for academic radiation oncologists in 2016 the mean h‐index was 14.5, and h‐index greater than 21 was correlated with senior faculty status versus junior.^11^

While the h‐index is an important objective metric, it has limitations and there are other metrics that can help quantify academic output. For example, the h‐index increases as citations accumulate during a career, and thus depends on a researcher's career length. The m‐quotient attempts to weigh the length of academic career and is defined as the h‐index divided by the number of years since the researcher's first publication.^11^ m‐quotient may allow better comparison of researchers across time, as h‐index is greatly affected by career length and not necessarily rate of academic productivity.^12^ Higher m‐quotient has been weakly correlated with senior faculty status for academic radiation oncologists,^11^ but the m‐quotient could still be an important indicator for promotion of mid‐career faculty. Another important metric of productivity in academic medicine is the history of extramural funding from the National Institutes of Health (NIH) as a principal investigator (PI).^13^

In this manuscript, we provide an analysis of the academic output of faculty from accredited radiation therapy physics (RTP) residency programs in the United States in 2020, using the total number of publications, h‐index, m‐quotient, and a history of being an NIH funded PI as metrics of research productivity.

## 2 METHODS

### 2.1 Departmental and faculty inclusion criteria

Departments with RTP residencies listed on the Commission on Accreditation of Medical Physics Education Programs (CAMPEP) website were included for analysis (https://www.campep.org/campeplstres.asp). The available departmental websites were accessed in December 2020 to determine the gender, highest academic degree, and academic rank or title of each RTP listed. Not all programs explicitly separate clinical faculty from research faculty or faculty from staff, so all RTP staff listed on departmental websites were included in this analysis. This included all RTP with either a doctorate or terminal master's degree. Designations as full professor, chairperson, chief, or director were classified as senior faculty. Associate professors, assistant professors, and clinical instructors as well as other faculty titles not fitting the previously mentioned categories were classified as junior faculty. While not necessarily a standard definition of junior faculty, this stratification was made for easy comparison to previously published metrics for radiation oncologists.^11^ No distinction was made between clinical, research, or tenure‐track faculty since these designations are not always easily determined from departmental websites.

### 2.2 Academic productivity data collection

For each RTP, the Scopus database (Elsevier BV, Amsterdam, The Netherlands) was queried to determine the h‐index, total number of publications, and year of first publication. Scopus currently has over 76 million records from journals, book series, and conference materials that have an International Standard Serial Number assigned to them. Because many conference abstracts are published in journals, the total publication count includes some peer‐reviewed abstracts. While the number of published conference abstracts is not indicative of research productivity, these cannot be easily filtered from the data. It also includes articles in press from more than 3850 journals.^14^, ^15^, ^16^ Therefore, the total publication number may seem high compared to a particular author's list of peer‐reviewed publications. The h‐index is not generally affected by the inclusion of abstracts, however, because most abstracts have few citations. Academic career duration was estimated as the year of a faculty's first publication in Scopus subtracted from the year 2020. The m‐quotient was calculated by dividing the Scopus h‐index by the academic career duration. Because of its popularity, a search was also performed for each RTP using Google Scholar (GS) (Google, LLC, Menlo Pak, CA) to obtain the h‐index.

The NIH Research Portfolio Online Reporting Tools (https://report.nih.gov/) query function was used to determine any history of NIH funding as a PI.

### 2.3 Statistical analysis

Descriptive statistics were calculated, including mean, SD, median, and interquartile range. h‐index and m‐quotient by academic rank and individual characteristics were also analyzed. Logistic regression analysis was used to generate predictive probability curves for professorial faculty ranks as a function of h‐index. The quality of the final regression was assessed for discrimination using the area under the receiver‐operator‐characteristic (ROC) curve (AUC). This analysis of the ROC curve serves as the model validation.^17^ The h‐index and m‐quotient data were not normally distributed; therefore, the Wilcoxon rank sum test with continuity correction was used for comparison of continuous variables and the Pearson χ^2^ test was used for categorical variables. For family‐wise error rate correction, the Bonferroni method was applied. This method, known for its conservative approach, would deter any false positives for trends affecting faculty promotion.^18^ Although a single comparison study may consider *p*‐values less than 5% significant, this threshold was adjusted to 0.3% given 15 comparisons over the course of the study.

## 3 RESULTS

A total of 1038 RTP were identified at 78 institutions with CAMPEP‐accredited residencies. Table 1 summarizes the overall group, along with gender characteristics and terminal degree. The mean h‐index for all academic RTP is 10.7, average m‐quotient is 0.66, and 10.5% have been an NIH PI. Women are under‐represented in academic medical physics in comparison to the US population; of the 1038 total physicists, 75.3% are male and 24.7% are female, while approximately 47% of the US workforce is female. This distribution is consistent with the American Association of Physicists in Medicine (AAPM) membership, which was 23.3% female in 2019, and is higher than the percentage of females earning bachelor's degrees in physics.^19^ RTP include 235 with terminal master's degrees and 803 with doctorates. Female physicists represent 30.2% of RTP with terminal master's degrees and 23% of RTP with doctorates. Not surprisingly, master's‐level physicists are skewed toward pure clinical positions, as suggested by statistically significant lower academic productivity metrics. Of the 1038 RTP from CAMPEP‐accredited programs, 141 (13.6%) have 0 publications in the Scopus database; 87 of the nonpublishers are RTP with terminal master's degrees while 54 have doctorates. Doctoral medical physicists on average have 58 publications and h‐index of 13.0, while master's‐level physicists on average have 7.3 publications and h‐index of 2.6. Both differences are statistically significant. Furthermore, 13.4% of doctoral physicists have a history as an NIH‐funded PI, while 0.4% of master's‐level physicists have been an NIH‐funded PI.

**TABLE 1:** Characteristics of RTP at CAMPEP‐accredited residency programs

	Total	Master's	Doctorate	*p*‐value^a^
N (%)	1038	235 (22.6)	803 (77.4)	
Gender, N (%)				
Male	782 (75.3)	164 (69.8)	618 (77)	<0.001
Female	256 (24.7)	71 (30.2)	185 (23)	
Senior Faculty, N (%)	172 (16.6)	3 (1.3)	169 (21)	<0.001
Career duration (years)				
Mean (SD)	13.5 (9.2)	8.1 (9.1)	15.1 (8.6)	<0.001
Median (IQR)	14 (7–20)	5 (0–14)	15 (9–21)	
Total publications				
Mean (SD)	46.7 (70)	7.3 (11.5)	58.2 (75.6)	<0.001
Median (IQR)	23 (5–57)	2 (0–10.5)	32 (12–74)	
Faculty with 0 publications, N (%)	141 (13.6)	87 (62)	54 (38)	<0.001
h‐index				
Mean (SD)	10.7 (11.5)	2.6 (3.5)	13.0 (12.0)	<0.001
Median (IQR)	7 (2–15)	1 (0–4)	10 (5–18)	
m‐quotient				
Mean (SD)	0.66 (0.56)	0.25 (0.32)	0.78 (0.56)	<0.001
Median (IQR)	0.6 (0.26–0.96)	0.14 (0.0–0.4)	0.74 (0.42–1.00)	
NIH funding, N (%)	109 (10.5)	1 (0.4)	108 (13.4)	<0.001

Abbreviation: IQR, interquartile range.

^a^ *p*‐value compares master's versus doctorate faculty using Wilcoxon test for continuous variables and Pearson test for categorical variables.

### 3.1 Academic productivity characteristics

Table 2 provides descriptive statistics for academic productivity metrics as a function of academic rank. The academic ranks recognized were instructor (or lecturer), assistant professor (or assistant faculty), associate professor (or associate faculty), professor, and director (or chair or chief). Any other designation was placed into a category of “no traditional academic rank.”

**TABLE 2:** Distribution of academic productivity metrics by academic rank

	No rank	Instructor	Assistant professor	Associate professor	Professor	Director
N	276	57	334	199	95	77
Gender, N (%)						
Male	203 (73.6)	38 (69.1)	238 (71.3)	158 (79.8)	78 (82.1)	66 (85.7)
Female	73 (26.4)	19 (34.5)	96 (28.7)	40 (20.2)	17 (17.9)	11 (14.3)
Career duration (years)						
Mean (SD)	8.6 (8.8)	7.7 (7.8)	10.9 (7.3)	17.8 (6.2)	24.3 (5.0)	22.4 (7.0)
Median (IQR)	7 (0–14)	6 (0–13)	11 (5–15)	18 (14–22)	26 (21–28)	24 (19–28)
No. of publications						
Mean (SD)	10.1 (15.6)	7.9 (10.9)	24.3 (31)	66.4 (47.8)	133.4 (94.0)	145.4 (127.7)
Median (IQR)	4 (0–13.3)	5 (0–10)	18 (7–31)	55 (35–82.8)	105 (73–171.5)	114 (43–203)
h‐index						
Mean (SD)	3.5 (4.6)	3.3 (4.4)	7.1 (7.0)	15.9 (8.5)	26.0 (13.1)	24.6 (16.2)
Median (IQR)	2 (0–5)	2 (0–4)	5 (3–9)	14.5 (10–20)	24.0 (17–33)	22 (11–36)
m‐quotient						
Mean (SD)	0.32 (0.36)	0.34 (0.39)	0.65 (0.62)	0.90 (0.43)	1.07 (0.47)	1.01 (0.57)
Median (IQR)	0.2 (0.0–0.5)	0.17 (0.0–0.5)	0.53 (0.3–0.84)	0.8 (0.64–1.08)	1.0 (0.79–1.31)	1.0 (0.57–1.23)
NIH funding, N (% per category)	0 (0)	0 (0)	10 (3)	27 (13.6)	40 (42.1)	32 (41.6)

Abbreviation: IQR, interquartile range.

All academic productivity metrics increase with increasing academic rank. On average, over a 7.7‐year academic career, instructors have produced 7.9 publications, with h‐index of 3.5 and m‐quotient of 0.32. On average, assistant professors have a 10.9‐year academic career, have produced 24.3 publications, have h‐index of 7.1, and m‐quotient of 0.65. On average, associate professors have a 17.8‐year academic career, have produced 66.4 publications, have h‐index of 15.9, and m‐quotient of 0.9. On average, full professors have a 24.3‐year academic career, have produced 133.4 publications, have h‐index of 26.0, and m‐quotient of 1.07. Additionally, 42.1% of full professors have been an NIH‐funded PI, while only 13.6% of associate professors and 3% of assistant professors have a history of NIH funding as a PI. For those in director positions, the average career duration, h‐index, m‐quotient, and percentage with NIH funding as a PI are all slightly lower than those for full professors.

**FIGURE 1:**
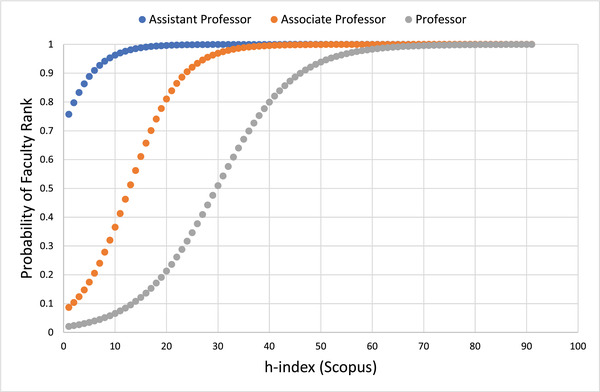
Predictive probability of faculty ranks as a function of Scopus Hirsch index

**FIGURE 2:**
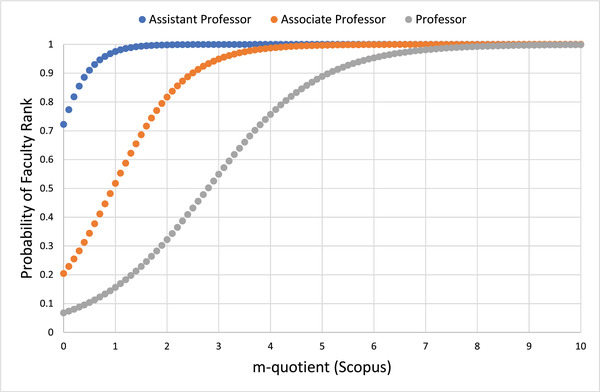
Predictive probability of faculty ranks as a function of m‐quotient calculated from Scopus data

Figure 1 shows predictive probability curves for faculty ranks of assistant, associate, and full professor, with the h‐index as the independent variable. Figure 2 shows similar curves with the m‐quotient as the independent variable. Table 3 summarizes the parameters used for the logistic regression. P, the probability of achieving a minimum rank of the tested professorship level follows the equation:

(1) $$P=\frac{e^{C_{1}+C_{2}X}}{1+e^{C_{1}+C_{2}X}}$$

where C_2_ is the index coefficient and C_1_ describes the intercept.

**TABLE 3:** Coefficients of estimation for logistic regression

		Index coefficient	Probability increase per index, %	*p*	Maximum AUC	AUC	Accuracy
h‐index (Scopus)	Assistant	0.23432 (0.03807)	26.41	<0.001	5.53	0.75	0.72 (0.68–0.75)
	Associate	0.20035 (0.01574)	22.18	<0.001	9.67	0.81	0.81 (0.78–0.84)
	Full Professor	0.13456 (0.01335)	14.40	<0.001	16.83	0.82	0.82 (0.79–0.85)
m‐index (Scopus)	Assistant	2.7209 (0.4370)	1419.40	<0.001	0.46	0.69	0.73 (0.69–0.76)
	Associate	1.4275 (0.1876)	316.83	<0.001	0.67	0.70	0.70 (0.66–0.73)
	Full Professor	0.9402 (0.1885)	156.05	<0.001	0.77	0.69	0.64 (0.60–0.68)

Abbreviations: AUC, area under the curve.

The increase in probability of a particular professorial designation per unit increase in the covariate, in this case the h‐index or m‐quotient, can be calculated from:

(2) $$\%\mathrm{Odds}\;\mathrm{increase}=(e^{C_{2}}-1)\times 100\%$$

The values of the h‐index and m‐quotient that maximize the AUC in an ROC analysis were determined. Using this methodology, the h‐index values that maximize AUC for designation as an assistant professor, associate professor, or full professor are 5.5, 9.7, and 16.8, respectively, and the corresponding m‐quotient values are 0.46, 0.67, and 0.77. While these values cannot be considered cutoff values for specific academic designations, the h‐index values in this model for associate and full professor have 81%–82% predictive accuracy.

### 3.2 Characteristics of RTPs with the highest h‐indices

Table 4 summarizes research productivity of the top 10% of medical physics academic faculty ranked by h‐index. This allows comparison with metrics previously published for radiation oncologists.^2^ The top 111 academic producers had an h‐index of 25 or higher. The mean h‐index in the group was 36.2, and the highest was 91. Additionally, 53.2% of the top 10% ranked by h‐index have been an NIH‐funded PI. The *p*‐values reported are for the comparison of junior to senior faculty for each parameter.

**TABLE 4:** Summary of top 10% by h‐index

	Total	Junior^a^	Senior^b^	*p* ^c^
N	111	33	88	
Gender, N (%)				
Male	94 (84.7)	28 (84.8)	66 (84.6)	0.5144
Female	17 (15.3)	5 (15.2)	12 (15.4)	
Career duration (years)				
Mean (SD)	25.1 (4.6)	21 (5.1)	26.9 (1.4)	<0.001
Median (IQR)	27 (23–28)	21 (18–25)	28 (26–28)	
No. of publications				
Mean (SD)	195.5 (104)	148.9 (67.6)	215.2 (110.6)	<0.001
Median (IQR)	178 (118–245)	137 (93–189)	190 (142.25–264.5)	
h‐index				
Mean (SD)	36.2 (11.6)	32.8 (10.0)	37.7 (12)	<0.001
Median (IQR)	33 (27–40)	29 (26–35)	36 (28–41.75)	
m‐quotient				
Mean (SD)	1.50 (0.60)	1.70 (0.70)	1.40 (0.4)	0.953
Median (IQR)	1.4 (1.13–1.7)	1.4 (1.29–1.86)	1.3 (1.11–1.58)	
NIH funding, N (% per category)	59 (53.2)	6 (18.2)	53 (67.9)	<0.001

Abbreviation: IQR, interquartile range.

^a^ Junior faculty includes staff, instructors, assistant professors, associate professors, and other faculty not otherwise specified.

^b^ Senior faculty includes full professors and directors.

^c^ *p*‐value compares senior versus junior faculty using Wilcoxon test for continuous variables and Pearson test for categorical variables.

The group was separated into junior and senior faculty. Of the top 10%, 88 are senior faculty and 33 are junior. All metrics are statistically higher for the senior faculty group than for the junior faculty group, except for gender and the m‐quotient, which is the same for both groups. There are possible explanations for the seemingly large number of junior faculty in the top 10%. Firstly, four of these researchers were in medical physics as a second career, and much of their academic output was in a field other than therapy physics. These individuals have very high h‐indices, but their academic rank is either assistant or associate professor. Additionally, one researcher is at an institution that does not have traditional faculty titles and was placed in the junior faculty group. Similar trends are evident when one examines the characteristics of the top quartile by h‐index (Table 5).

**TABLE 5:** Summary of top 25% by h‐index

	Total	Junior^a^	Senior^b^	*p* ^c^
N	279	148	131	
Gender, N (%)				
Male	230 (82.4)	121 (84.8)	109 (83.2)	0.3761
Female	49 (17.6)	27 (18.2)	22 (16.8)	
Career duration (years)				
Mean (SD)	22.1 (5.7)	19.6 (5.5)	25.0 (4.6)	<0.001
Median (IQR)	23 (18–28)	19 (16–23.3)	27 (23–28)	
No. of publications				
Mean (SD)	126.4 (92.9)	87.6 (53.2)	170.3 (107.7)	<0.001
Median (IQR)	95 (64.5–159.5)	73 (53–100.3)	144 (93.5–210.5)	
h‐index				
Mean (SD)	25.8 (11.4)	21.7 (8)	30.5 (12.9)	<0.001
Median (IQR)	22 (18–30.5)	19.5 (16–24)	27 (21–36.5)	
m‐quotient				
Mean (SD)	1.2 (0.5)	1.20 (0.60)	1.2 (0.4)	0.176
Median (IQR)	1.1 (0.88–1.39)	1.1 (0.84–1.38)	1.1 (0.93–1.42)	
NIH funding, N (%)	92 (33.0)	26 (17.6)	66 (50.4)	<0.001

Abbreviation: IQR, interquartile range.

^a^ Junior faculty includes staff, instructors, assistant professors, associate professors, and other faculty not otherwise specified.

^b^ Senior faculty includes full professors and directors.

^c^ *p*‐Value compares senior versus junior faculty using Wilcoxon test for continuous variables and Pearson test for categorical variables.

Additionally, it is seen that there is a large disparity in the history of NIH funding between the senior and junior faculty in this cohort, with 68% of senior faculty having been an NIH‐funded PI compared to only 18% of junior faculty. These results compare favorably with previously published data showing increases in NIH funding for older AAPM members.^20^ While disparities exist, a significant correlation between NIH PI status and faculty promotion was not evident.

### 3.3 Gender characteristics of senior faculty

We further examined the gender characteristics of senior faculty (Table 6). Of the 172 senior RTP, 144 are male (83.7%) and 28 are female (16.3%). This is statistically different than the overall composition of RTP faculty, which is approximately 25% female. The h‐index was slightly higher for male senior faculty and approached statistical significance. The history of having been an NIH‐funded PI was statistically higher for male faculty. These results corroborate the comprehensive study on NIH research funding for AAPM members by Whelan et al., showing that the number of grants awarded to different genders within AAPM are disparate, with 11% held by females in a 22% female population.^20^ Similarly, our study found that 13.4% of female PIs held NIH grants in a 24.7% female population.

**TABLE 6:** Gender characteristics of senior faculty^a^

	Total	Men	Women	*p* ^b^
Senior academic rank, N (%)	172 (16.6)	144 (83.7)	28 (16.3)	<0.001
Career duration (years)				
Mean (SD)	23.5 (6.0)	23.6 (6.1)	23.0 (5.7)	0.226
Median (IQR)	25 (20–28)	25 (20–28)	24 (19.8–28)	
No. of publications				
Mean (SD)	138.8 (110.2)	146.4 (116.8)	99.7 (53.2)	0.006
Median (IQR)	108.5 (65.5–187)	114 (65–193)	92 (62.5–128.5)	
h‐index				
Mean (SD)	25.4 (14.6)	26.0 (15.3)	22.4 (9.6)	0.095
Median (IQR)	23 (15–34)	23 (15–35.3)	21 (16.5–27.3)	
NIH funding, N (%)	72 (41.9)	62 (43.1)	10 (37.5)	0.008

Abbreviation: IQR, interquartile range.

^a^ Senior faculty are defined as full professors or directors.

^b^ *p*‐value compares men versus women senior faculty using Wilcoxon test for continuous variables and Pearson test for categorical variables.

### 3.4 Comparison of h‐index in GS and Scopus

**FIGURE 3:**
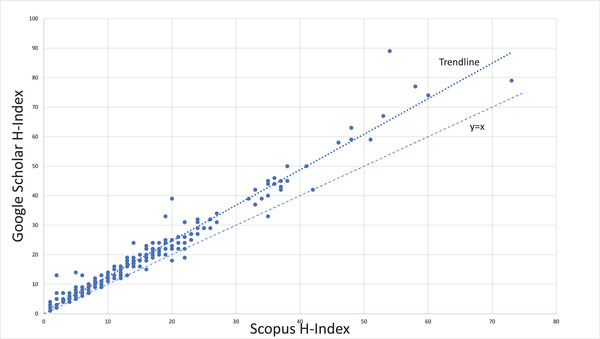
Hirsch index (h‐index) in Google Scholar versus Scopus for the 214 academic therapy physicists who had data in both systems; Google Scholar generally reports a larger h‐index than Scopus

**FIGURE 4:**
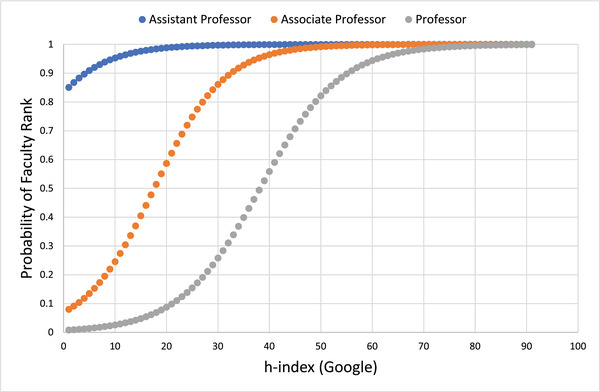
Predictive probability of faculty ranks as a function of Google Scholar Hirsch index

While GS is a convenient tool for literature searches and determining citation impact, individual investigators need to have created a GS page to determine their personal bibliometric metrics. GS pages were found for only 214 of the 1038 RTP in this study. The GS h‐index is consistently higher than the Scopus h‐index; linear regression finds that the GS h‐index is 1.27 times higher than the Scopus h‐index (Figure 3). It has been shown that GS overestimates citations due to inclusion of non‐peer‐reviewed publications and duplicate records.^21^, ^22^, ^23^, ^24^, ^25^ It can still be a useful tool for gauging academic productivity when used with the appropriate benchmarks, and Figure 4 shows the predictive probability for faculty ranks as a function of the GS h‐index. Further comparisons are discussed in the work by Bar‐Ilan.^26^ For the purposes of our study, we focused on Scopus h‐indices because of its availability for all published researchers.

## 4 DISCUSSION

Many factors are considered in hiring, tenure, and promotion decisions, including clinical proficiency, teaching evaluations, institutional service, service to the profession, and academic achievement. When assessing research achievement alone, many faculty contributions are considered, including extramural funding, scientific presentations, organizing and hosting workshops, participation in clinical trials, task group membership, and publication record. Historically, a researcher's total number of publications and/or citations has been used to assess the publication record. The h‐index has become increasingly recognized as an objective indicator of academic productivity, since it provides a more complete overview of the author's publication record and overall research impact.^1^, ^2^, ^7^, ^11^ Additional metrics have also been used to supplement the h‐index. In this study, increased h‐index, m‐quotient, and career duration, along with a history of NIH funding as a PI, were all associated with increased academic rank for RTP associated with CAMPEP‐accredited residency programs. Academic department directors have a slightly lower average h‐index than full professors do. There are many possible reasons for this, including the possibility that administrative duties displace research activities, leading to lower research output.

Over 13% of RTPs associated with CAMPEP‐accredited programs have published zero publications. The majority of these were physicists with terminal master's degrees, who are often in primarily clinical roles. Conversely, among the top 10% of RTP ranked by h‐index, all were doctoral‐level physicists and 88 (79%) were senior faculty. A gender discrepancy was found, with just 15% of these top RTPs (ranked by h‐index) being women.

An interesting point to note is that the h‐indices for RTP tend to be lower than those for physician faculty in radiation oncology. Compared with the data in Zhang et al., the radiation oncology faculty's h‐index average of 14.5 is higher than RTP's h‐index average of 10.7. This disparity remains when one considers senior faculty only, where the average h‐index is 32.3 for physician faculty compared to 25.4 for physics faculty.^11^ This could be partially explained by the larger readership of clinical journals compared to technical journals. Comparison of the 2‐year journal impact factors of some common journals in radiation oncology and medical physics objectively demonstrates the higher impact of clinical innovation compared to technical innovation—*International Journal of Radiation Oncology Biology Physics* (5.86), *Medical Physics* (3.32), *Practical Radiation Oncology* (2.95), and *Journal of Applied Clinical Medical Physics* (1.68).^27^ Having a history of being a PI with the NIH, however, is similar for senior medical physics faculty in comparison to physician colleagues in radiation oncology; 42.1% of RTP full professors and 41.6% of RTP directors have been PIs on NIH grants, compared to 32.3% of radiation oncology professors and 38.7% of department chairs. Outside of the most senior RTP, however, NIH funding is rare enough to be considered an anomaly, with only 13.6% of RTP associate professors having served as PIs. The relative scarcity of RTPs successfully competing for NIH funding makes this a questionable metric for assessing RTP. The data are included here, however, because obtaining extramural funding, and NIH funding specifically, is considered in promotion and tenure decisions at some academic institutions.

Gender disparities have been found to exist in many medical specialties,^11^, ^19^, ^28^, ^29^, ^30^, ^31^ and our study showed similar results with men having longer career durations, more publications, and higher h‐indices than women. However, in our study these results are somewhat skewed because of a disproportionate number of women who have terminal master's degrees and who are nonpublishers. While it still exists, the gender disparity in academic productivity is much less when considering only senior faculty, and the disparity is even less when considering only the top 10% and top quartile of academic producers using the h‐index. However, the large gender disparity in RTP leadership positions seen in other studies^19^ was also evident in our study. Further study is warranted to better understand the reasons for these disparities and develop strategies to increase the gender diversity in the RTP workforce and leadership.^32^

Our study includes a large and comprehensive sample size of academic RTP and provides important benchmarking data for RTP. There are limitations, however. We relied on the CAMPEP‐listed departmental websites to determine RTP affiliation and title. If these are not updated regularly, the results could be incomplete or incorrect. Throughout the course of data collection, separate staff performed several spot checks to ensure the accuracy of the data. Furthermore, RTP represents a very heterogeneous population with great diversity in terminal degree and responsibilities. Many RTPs, even at academic institutions, focus on clinical service and teaching, with little or no responsibility for the research mission of their institution. Since departmental websites do not necessarily delineate responsibilities, however, we made no attempt to stratify staff members into different bins such as tenure track, research track, clinical track, or staff. Including those with no research responsibilities skews the analysis downward for the overall population, However, any physicist who had no academic title listed was grouped into the “no rank” and “junior” categories in our analysis. Hence, this analysis provides valid benchmarks for those with traditional academic titles and those classified as senior faculty. We relied primarily on Scopus and the NIH online reporting tools for the data collection, and there are potential errors, including the potential to incorrectly attribute a researcher's work to another individual with the same name. We also did not correct for the potential for name changes related to maiden name, married name, nicknames, or initials. These inconsistencies could also affect the results, and many of these can disproportionately affect women. For example, one study showed that female faculty in radiation oncology often published under more than one last name, and this affected their h‐index in 8% of cases.^31^ Scopus is the largest curated citation database, and has consistently improved its coverage and author profiles.^14^, ^15^, ^16^ It is reasonable to expect that bibliometric databases will continue to improve. Furthermore, it is recommended that individual authors review their profiles to make sure that they are accurate and complete. This will additionally improve the accuracy of the databases and any future benchmarks generated from these databases. There are also limitations to the academic metrics studied. For example, the h‐index grows over time, and faculty early in their career will have a low h‐index. The m‐quotient was used to account for this, but it is a much weaker predictor of academic success than the h‐index. Viewing Table 3, one may note that the accuracies for the h‐index logistic model predicting the ranks of associate and full professorship are higher than the accuracies for the m‐index logistic model. There is confidence in this assessment because the SDs do not overlap, with a minimum expected advantage of 0.05 and 0.11 in predictive power for the h‐index model. Additionally, there is error introduced by using time from first publication to determine career duration, as many RTP have published papers during their training. This affects both the career duration and m‐quotient.

## 5 CONCLUSION

This report compiles objective metrics describing research productivity for RTPs associated with CAMPEP‐accredited residency programs. These metrics include total number of publications, the h‐index, m‐quotient, and history of obtaining NIH funding as a PI. While these data alone cannot be used to make any personnel decisions, they can be used as an easily obtained, quantifiable, and objective benchmark describing a small portion of an academic RTP's overall productivity. To our knowledge, this is the first study to compile these data, which may be of interest to faculty preparing for promotion, and also to institutional leadership.

## CONFLICT OF INTEREST

The authors declare no conflict of interest.

## AUTHOR CONTRIBUTIONS

Conception and design of the study: Sanford L. Meeks, Patrick Kelly, and Amish P. Shah. Data acquisition: Sanford L. Meeks. Statistical analysis and figure generation: Michael H. Shang. Preparation of draft manuscript: Sanford L. Meeks. Interpretation of the data, revisions and critical feedback, and final approval: all authors.
